# Deregulated FADD expression and phosphorylation in T-cell lymphoblastic lymphoma

**DOI:** 10.18632/oncotarget.11370

**Published:** 2016-08-18

**Authors:** José L. Marín-Rubio, María C. de Arriba, María A. Cobos-Fernández, Laura González-Sánchez, Inmaculada Ors, Isabel Sastre, José Fernández-Piqueras, María Villa-Morales

**Affiliations:** ^1^ Centro de Biología Molecular Severo Ochoa (CBMSO), Consejo Superior de Investigaciones Científicas-Universidad Autónoma de Madrid (CSIC-UAM), Madrid, Spain; ^2^ IIS-Fundación Jiménez Díaz, Madrid, Spain; ^3^ Universidad Autónoma de Madrid, Departamento de Biología, Madrid, Spain; ^4^ Universidad Carlos III, Departamento de Bioingeniería, Madrid, Spain; ^5^ Centro de Investigaciones Energéticas, Medioambientales y Tecnológicas (CIEMAT), Madrid, Spain; ^6^ Centro de Investigaciones Biomédicas en Red de Enfermedades Raras (CIBERER), Valencia, Spain

**Keywords:** T-cell lymphoblastic lymphoma, Fas-associated death domain protein (FADD), expression and phosphorylation, tumor aggressiveness, prognostic marker

## Abstract

In the present work, we show that T-cell lymphoblastic lymphoma cells exhibit a reduction of FADD availability in the cytoplasm, which may contribute to impaired apoptosis. In addition, we observe a reduction of FADD phosphorylation that inversely correlates with the proliferation capacity and tumor aggressiveness. The resultant balance between FADD-dependent apoptotic and non-apoptotic abilities may define the outcome of the tumor. Thus, we propose that FADD expression and phosphorylation can be reliable biomarkers with prognostic value for T-LBL stratification.

## INTRODUCTION

Precursor T-cell lymphoblastic neoplasms are aggressive hematological malignancies, derived from immature thymocytes in various differentiating stages. When they manifest as a mass lesion in the thymus/anterior mediastinum or in lymph nodes, with less than 25% marrow blasts, they are called T-cell lymphoblastic lymphoma (T-LBL) [1]. Molecular genetics of T-LBL are not well characterized, mainly due to the scarcity of samples [2].

Cancer cells frequently become resistant to a possible apoptotic insult mediated by FAS. Such resistance may be acquired by means of different mechanisms [3, 4]. In a recent study, we observed that 35% of human T-LBL cases exhibited reduced levels of Fas-associated death domain protein (FADD), suggesting that FADD reduction would be a frequent mechanism whereby FAS-mediated apoptotic signaling would be affected in this type of tumor [5]. FADD reduction has been also observed in tumor types like human non-small cell lung cancer [6], hepatocellular carcinoma [7] or thyroid adenoma/adenocarcinoma [8].

Although apoptosis is the canonical role for FADD, evidence compiled in the recent years indicate that FADD may also contribute to survival, cell cycle progression and cell proliferation, depending on its phosphorylation status and subcellular localization [9]. Such role for FADD in non-apoptotic functions is especially relevant in T cells at early stages of hematopoiesis [10, 11]. Moreover, thymopoiesis is partially defective in FADD-mutant mice [12]. FADD has been shown critical for regulating apoptosis of T-cell progenitors at the pre-TCR checkpoint, but also, it mediates signals required for efficient proliferation during transition from the CD4^−^CD8^−^ double-negative (DN) to the CD4^+^CD8^+^ double-positive (DP) stage [13, 14]. Furthermore, FADD deficiency results in problems at multiple cell cycle checkpoints, not only inhibiting the entry of activated T cells into the cell cycle, but also blocking the cell cycle progression of already proliferating cells in G1 phase [15, 16]. Altogether, this indicates that FADD may represent a tumor suppressor with positive and negative effects on T cell growth [14].

FADD ability to promote the progression of T cells through the cell cycle seems to depend on its phosphorylation status [17, 18]. FADD phosphorylation occurs at Ser194 in humans and its murine equivalent Ser191 [19]. FADD phosphorylation might be biphasic in primary T cells, occurring during G0 to G1 followed by a dephosphorylation event during G1 to S and subsequent re-phosphorylation at G2/M [20]. Mice transgenic for the phosphomimetic mutation of FADD (Ser191Asp) showed analogous characteristics to FADD-deficient mice [9, 17]. Interestingly, the subcellular localization of FADD seems to be associated with this modification, specifically S194/S191-P-FADD would be mainly nuclear [18]. However, the exact mechanism whereby nuclear FADD exerts its non-apoptotic function is not clear and the contribution of FADD subcellular localization to its function remains a recurrent issue. The simple hypothesis is that sequestration of FADD in the nucleus prevents its interaction with death receptors. But FADD could also bind other proteins to form a functional transcription factor complex to regulate gene transcription and activate survival mechanisms [9].

Several kinases have been proposed as responsible for FADD phosphorylation: HIPK3, which is induced by JNK, seems to promote FADD phosphorylation in prostate cells [21]. PKCζ has been described to phosphorylate FADD in certain cell types [22]. A model has been proposed [23], according to which CK1α and FADD colocalize on the mitotic spindle early in mitosis, resulting in phosphorylation of FADD at Ser194. It has been also reported the interaction between FADD and PLK1 [24, 25], apparently in a G2/M-specific manner [26]. Finally, DUSP26 has been recently described as a phosphatase responsible for FADD dephosphorylation in breast cancer cells [27].

FADD phosphorylation has been analyzed in numerous solid tumors, however very limited and heterogeneous results are available in hematological cancers [28, 29]. Thus, whether alterations in FADD expression and phosphorylation are involved in T-cell lymphoblastic lymphoma kept an unresolved question.

Our study presents, for the first time, with a comprehensive study of FADD levels, phosphorylation status, regulators of FADD phosphorylation, FADD subcellular localization and function in T-cell lymphoblastic lymphoma. According to the results, FADD phosphorylation is altered in T-LBL, probably due to the combined action of several mechanisms: reduced FADD levels, reduced levels of HIPK3 and CK1α kinases and elevated levels of DUSP26 phosphatase. Altogether, FADD reduction may result in diminished apoptosis of tumor thymocytes. Moreover, two distinct sub-groups of T-LBL can be established based on S191-P-FADD levels, which correlate with different proliferation capacities and tumor aggressiveness. Thus, we propose that FADD expression and its phosphorylation can be reliable biomarkers with prognostic value for T-LBL stratification.

## RESULTS

### FADD and phospho-FADD levels are reduced in mouse T-LBL

**Figure 1:**
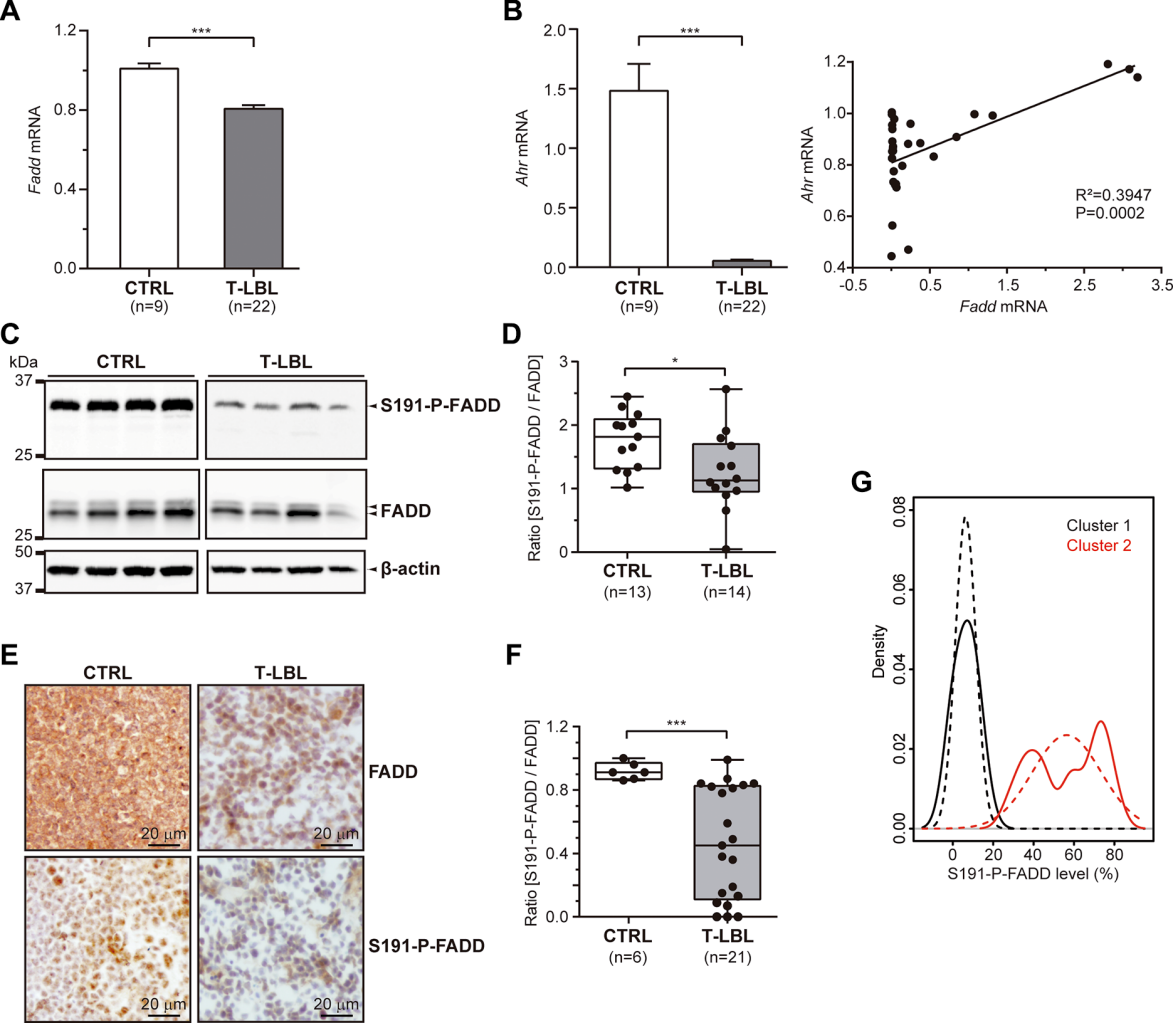
FADD and P-FADD levels in T-LBL (**A**, **B**) *Fadd* (A) and *Ahr* (B) mRNA levels were determined in healthy thymuses (CTRL) and T-cell lymphoblastic lymphoma samples (T-LBL) by quantitative RT-PCR. The results were normalized using the 2^−ΔΔC^_T_ method, referring *Fadd* or *Ahr* expression to those of *G6pd* and *Hprt*, and referenced to the control group. (**C**–**F**) Total FADD protein and S191-P-FADD levels were determined in CTRL and T-LBL groups by Western blot (C, D) and Immunohistochemistry (E, F). Representative images are shown for the WB (C) and IHC (E) experiments. WB images in (C) are cropped in favor of conciseness. The box-and-whisker plot analyses of the ratio [S191-P-FADD/FADD] for all the samples are shown for the WB (D) and IHC (F) experiments, indicating the statistical significance of the comparisons. (**G**) Kernel density plot showing T-LBLs density for S191-P-FADD levels (continuous line) and standard normal distribution of each cluster (dashed line). *P < 0.05. ***P ≤ 0.001.

We studied the levels of *Fadd* mRNA by quantitative RT-PCR in 9 healthy thymuses and 22 T-LBL samples from C57BL/6J mice. A significant reduction was observed in T-LBLs (fold-change = 20%, *P <* 0.0001), compared with the control group (Figure 1A).

We discarded the presence of mutations in the *Fadd* promoter sequence of T-LBL samples (genomic coordinates chr7:144581400-144582801 from Ensembl Genome Browser), which might have affected transcription factors binding (data not shown). Also, an *in-silico* analysis of this region showed the most relevant transcription factors binding sites, as predicted by SABiosciences' Text Mining Application and the UCSC Genome Browser (http://www.sabiosciences.com/chipqpcrsearch.php?species_id=1&factor=Over+200+TF&gene=FADD&nfactor=n&ninfo=n&ngene=n&B2=Search). We counted on preliminary evidence from RNA-sequencing data (unpublished), which indicated that one of those transcription factors, *Ahr*, exhibited a significant downregulation in all the T-LBL samples, in comparison with healthy thymuses. We validated these results by means of quantitative RT-PCR (Figure 1B). Interestingly, a regression analysis revealed a significant correlation between *Fadd* and *Ahr* mRNA levels in these samples.

At the protein level, total FADD and S191-P-FADD were studied by Western blot in whole protein extracts of thymocytes from 13 healthy thymuses and 14 T-LBL samples (Figure 1C, 1D). The statistical analysis after densitometry and β-actin normalization revealed a significant reduction of S191-P-FADD levels in T-LBLs (*P* = 0.019), expressed as the ratio [S191-P-FADD/FADD] (Figure 1D). This reduction was not due to the presence of mutations, as it was corroborated by *Fadd* cDNA sequencing (data not shown). We confirmed these results by immunohistochemistry (IHC) in tissue sections from 6 healthy thymuses and 21 T-LBL samples (Figure 1E, 1F), which also revealed a significant reduction of the ratio [S191-P-FADD/FADD] in tumors (*P <* 0.001) (Figure 1F).

**Figure 2:**
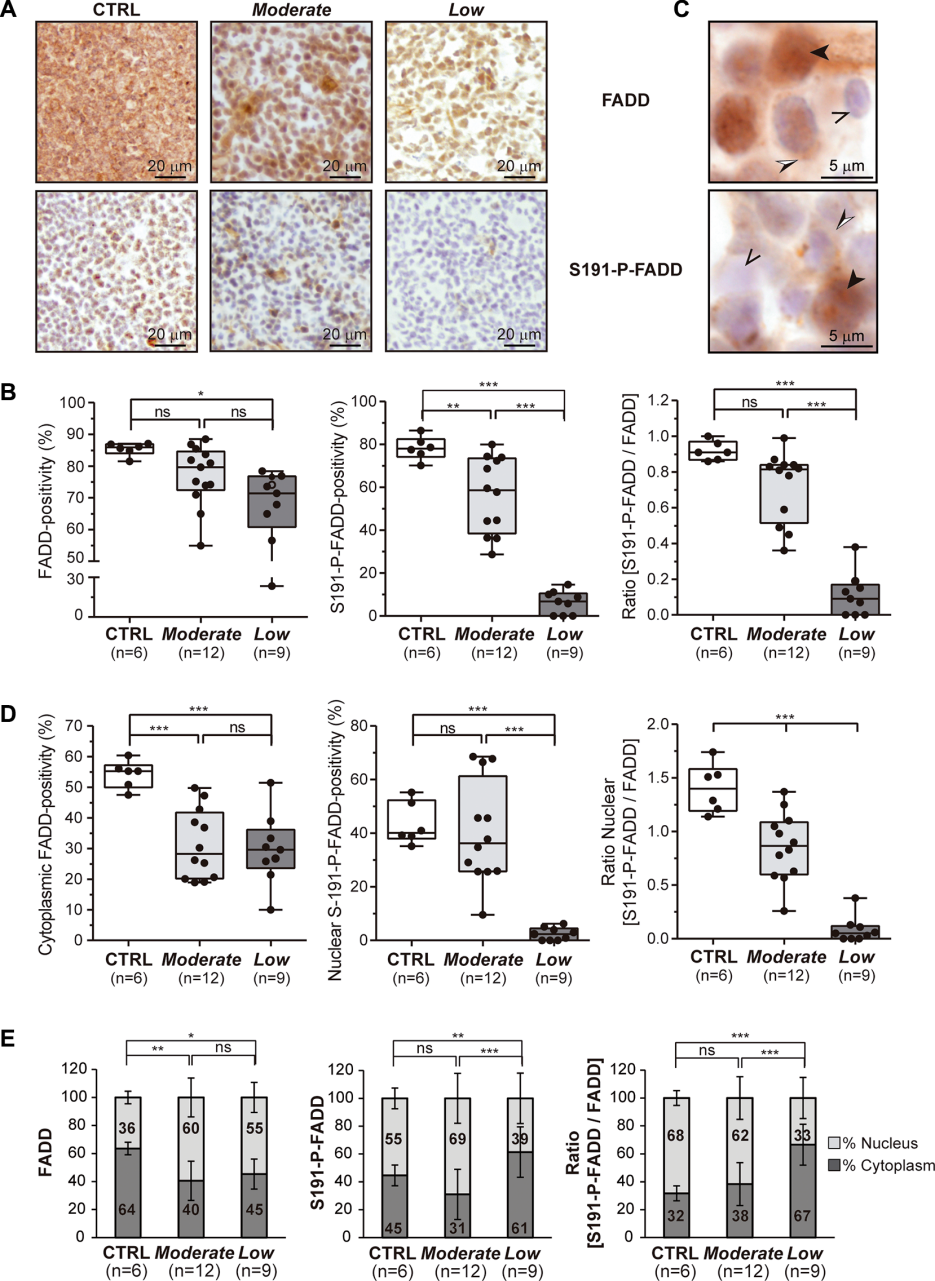
Stratification of T-LBL and subcellular localization of FADD and P-FADD (**A**, **B**) Total FADD protein and S191-P-FADD levels determined by IHC are shown for the so-called *Moderate* and *Low* T-LBL sub-groups, in comparison with the control group (CTRL). (A) Representative images are shown for each group. (B) The box-and-whisker plot analyses of total FADD, S191-P-FADD and the ratio [S191-P-FADD/FADD] for all the samples are shown, indicating the statistical significance of the comparisons. (**C**) To illustrate the subcellular localizations of FADD and S191-P-FADD, representative images acquired at 100 × magnification are shown. The black arrowheads illustrate cells with nuclear positivity, the black and white arrowheads illustrate cells with cytoplasmic positivity, and the open arrowheads illustrate negative cells. (**D**) The box-and-whisker plot analyses of cytoplasmic total FADD, nuclear S191-P-FADD and the nuclear ratio [S191-P-FADD/FADD] for all the samples are shown, indicating the statistical significance of the comparisons. (**E**) The relative distributions nucleus:cytoplasm of total FADD, S191-P-FADD and the ratio [S191-P-FADD/FADD] are represented for each group in bar charts, indicating the statistical significance of the comparisons.*ns*, not significant; *P < 0.05; **P < 0.01; ***P ≤ 0.001.

Notably, a considerable inter-tumor heterogeneity regarding FADD and – particularly - S191-P-FADD levels was observed among the T-LBL samples. We performed a Kernel density plot, which showed a skewed distribution of the samples in two clusters with moderate and low levels of S191-P-FADD positivity by IHC, compared with the control group (Figure 1G). These clusters define two T-LBL sub-groups, which will be named *Moderate* and *Low*, with reference to their levels of S191-P-FADD. Interestingly, the comparison according to this sub-classification revealed that the reduction of total FADD levels was statistically significant between the control group and the *Low* T-LBL sub-group (*P* = 0.012), but not between the two T-LBL sub-groups (Figure 2B). The percentage of S191-P-FADD-positive cells, obtained from the IHC experiments, revealed significant differences in all the comparisons (*P ≤* 0.01) and the ratio [S191-P-FADD/FADD] resulted significantly diminished in the *Low* T-LBL sub-group, both compared with the control group (*P* < 0.001) and with the *Moderate* T-LBL sub-group (*P <* 0.001) (Figure 2A, 2B).

### FADD sub-cellular localization in mouse T-LBL

The sub-cellular localizations of FADD and S191-P-FADD were also analyzed in control and T-LBL samples by IHC (Figure 2C–2E). Both the *Moderate* and *Low* T-LBL sub-groups exhibited a significant reduction of cytoplasmic FADD, compared with the controls (*P ≤* 0.001), but no significant difference existed between them (*P* = 1.000) (Figure 2D).

Besides, nuclear S191-P-FADD positivity showed no significant difference between the control group and the *Moderate* group (*P* = 1.000). However, the reduction in the *Low* group was statistically significant in both comparisons (*P <* 0.001) (Figure 2D). The ratio nuclear [S191-P-FADD/FADD] resulted significantly diminished both in the *Moderate* and *Low* T-LBL sub-groups, in comparison with controls (*P <* 0.001), and also between them (*P <* 0.001) (Figure 2D). If we compared the relative distribution nucleus/cytoplasm between the groups, interesting conclusions emerged (Figure 2E). The distribution for total FADD in *Moderate* and *Low* T-LBLs differed from that of the control group, with total FADD significantly decreasing in the cytoplasm of both T-LBL sub-groups (*P* = 0.002 and *P* = 0.025, respectively). Regarding S191-P-FADD, the relative distribution in the *Moderate* T-LBL sub-group was similar to that of the control group (*P* = 0.376), while the *Low* group presented with a significant reduction in the nucleus (*P* = 0.006). When expressed as the ratio [S191-P-FADD/FADD], we observed that the phosphorylated form of FADD was predominant in the nucleus of control thymocytes, while it became progressively redistributed in the thymocytes of the *Moderate* group (*P* = 1.000), but especially in the *Low* T-LBL group (*P ≤* 0.001).

### Apoptosis is reduced in T-LBL samples

**Figure 3:**
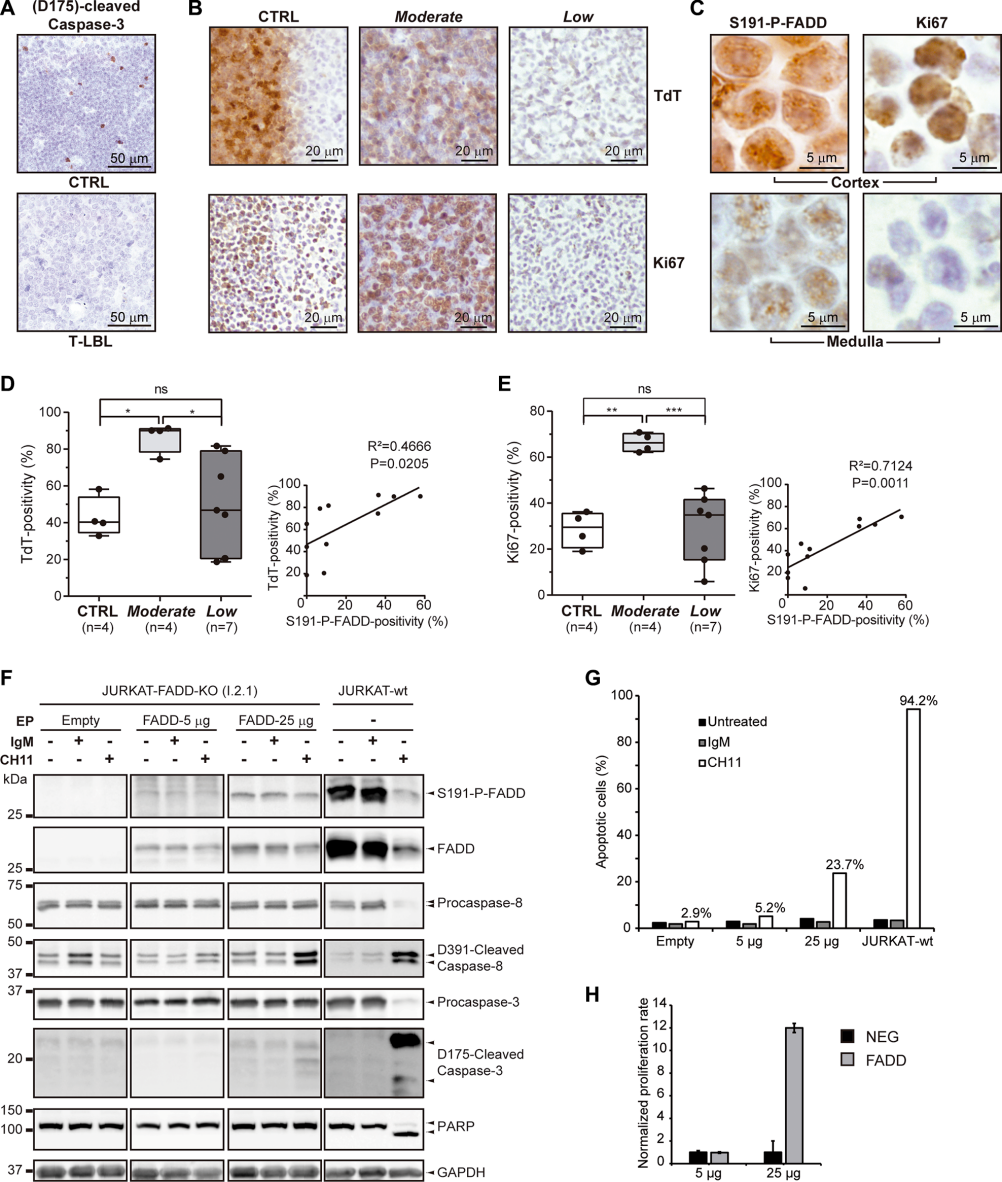
Apoptosis, tumor and proliferation markers in T-LBL (**A**) (D175)-cleaved-Caspase-3 levels are determined by IHC in healthy thymuses (CTRL) and T-LBL samples, as a measure for apoptosis. Representative images are shown for each group. (**B**, **D**, **E**) Terminal deoxynucleotidyl transferase (TdT) and Ki67 levels are determined by IHC in healthy thymuses (CTRL) and *Moderate* and *Low* T-LBL samples, as a measure for the T-LBL-characteristic blast presence and for proliferation, respectively. (B) Representative images are shown for each group. The box-and-whisker plot analyses of TdT (D) and Ki67 (E) percentages of positive cells for each group are shown, indicating the statistical significance of the comparisons. Also, their correlation with S191-P-FADD positivity are determined by regression analysis, indicating the coefficient of determination (R^2^) and the *P value*. (**C**) To illustrate the differential staining of S191-P-FADD in the cortex and medulla of healthy thymuses, representative images acquired at 100 × magnification are shown. (**F**, **G**) The apoptotic capacity was evaluated by WB in a FADD-reconstitution assay of I 2.1 FADD-deficient JURKAT cell line (JURKAT-FADD-KO). As a positive control, the parental FADD-wild type A3 JURKAT cell line is used (JURKAT-wt). As a negative control, I 2.1 cells were electroporated (EP) with the negative vector EX-NEG-Lv225. I 2.1 cells were electroporated with 5 or 25 μg of EX-V0108-Lv225 vector (FADD-5 μg and FADD-25 μg, respectively). Each condition was either left untreated (−) or treated for 24 h with irrelevant IgM isotype control antibody (IgM) or with agonist anti-FAS antibody (CH11). An aliquot was used for protein extraction and specific immunodetection of Caspases-8 and 3 activation and PARP proteolysis (F) and another was analyzed for Annexin V / 7-AAD staining by flow cytometry (G). WB images in (F) are cropped in favor of conciseness. (**H**) The proliferative capacity was evaluated in the same FADD-reconstitution assay. The normalized proliferation rates of the FADD-5 μg and FADD-25 μg conditions (FADD), and their corresponding EX-NEG-Lv225 electroporations (NEG), are represented in a bar chart, expressed as the ratio [number of cells at 72 h post-EP / number of cells at 72 h post-EP with the corresponding EX-NEG-Lv225 condition]. *ns*, not significant; *P < 0.05; **P < 0.01; ***P ≤ 0.001.

The IHC analysis of active caspase-3 as a marker for apoptosis revealed a striking reduction in the percentage of positive cells in 38.1% (8/21) of the T-LBL samples analyzed, in comparison with controls (*n* = 9) (Figure 3A). The T-LBL samples shown to be negative for active caspase-3 belonged either to the so-called *Moderate* or *Low* groups. This is consistent with the observation in Figure 2D that cytoplasmic FADD positivity was significantly reduced in both groups, compared with the controls, but no differences were found between them.

### The “Moderate” and “Low” T-LBL sub-groups present different blast numbers and proliferation rates

The presence of blast cells and the proliferation rate were estimated by TdT and Ki67 immunostaining, respectively (Figure 3B, 3D and 3E). In healthy thymuses (*n* = 4), TdT was typically distributed asymmetrically, being the cortex highly positive and the medulla fully negative. Likewise, Ki67 was more abundant in the cortex, and principally localized in the outer part, adjoining the capsule around the thymus. This correlated with S191-P-FADD distribution, which presented with a majority of S191-P-FADD-staining in the outer cortex, whereas the positivity in medulla was weaker (Figure 3C). Interestingly, nearly all the positive cells in the outer cortex showed predominantly nuclear S191-P-FADD-staining.

As expected in T-LBL samples, there was no longer a clear demarcation of cortex and medulla (Figure 3B). Notwithstanding, the blast percentage (Figure 3D) and proliferation rate (Figure 3E) were significantly higher in the *Moderate* T-LBL sub-group, compared both with the control group (*P* = 0.024 and *P* = 0.002, respectively) and with the *Low* T-LBL sub-group (*P* = 0.038 and *P ≤* 0.001), whereas no statistical difference was observed between the control group and the *Low* T-LBL sub-group (*P* = 1.000). Interestingly, a regression analysis revealed significant correlations between TdT (Figure 3D) or Ki67 (Figure 3E) and S191-P-FADD positivities, supporting the stratification of T-LBLs into *Moderate* and *Low* sub-groups.

Our next step was to determine the levels of FADD and P-FADD that are able to exert apoptotic and non-apoptotic functions in the cell (Figure 3F–3H). We reconstituted FADD expression in the I 2.1 FADD-deficient JURKAT cell line with 5 μg and 25 μg of FADD EX-V0108-Lv225 construct. Forty-eight hours after electroporation, the transfectants exhibited low and moderate P-FADD levels, respectively, compared with the parental FADD-wild type A3 JURKAT cell line. Upon apoptosis-induction with agonist anti-FAS antibody (CH11), only the parental FADD-wild type A3 JURKAT cell line showed an efficient execution of apoptosis, measured by PARP proteolysis (Figure 3F) and Annexin V/7-AAD staining (Figure 3G). None of the transfectants exhibited PARP proteolysis (Figure 3F); however, interesting differences were observed between them. The FADD-5 μg transfectant did not show any activation of the caspase cascade upon CH11-induction (Figure 3F), whereas the FADD-25 μg transfectant exhibited an apparent activation of Caspase-8 and a subtle but evident activation of Caspase-3 (Figure 3F). Furthermore, 23.7% of the cells in this condition exhibited early apoptosis measured as Annexin V^+^/7-AAD^−^, compared with 5.2% of cells in the FADD-5 μg condition (Figure 3G). Altogether, these results indicate that the increment in FADD levels leads to functional consequences, but suggest the presence of a threshold below which the cell is not able to execute generalized apoptosis.

On the other hand, the cell growth was determined at 72 hours (Figure 3H) and we found that the FADD-5 μg transfectant did not show any difference in terms of proliferation, compared with its negative counterpart. On the contrary, the FADD-25 μg transfectant had proliferated significantly more. This indicates that the increment in FADD levels leads to functional consequences different from apoptosis.

### The “Moderate” tumors are more aggressive than the “Low” tumors

**Figure 4:**
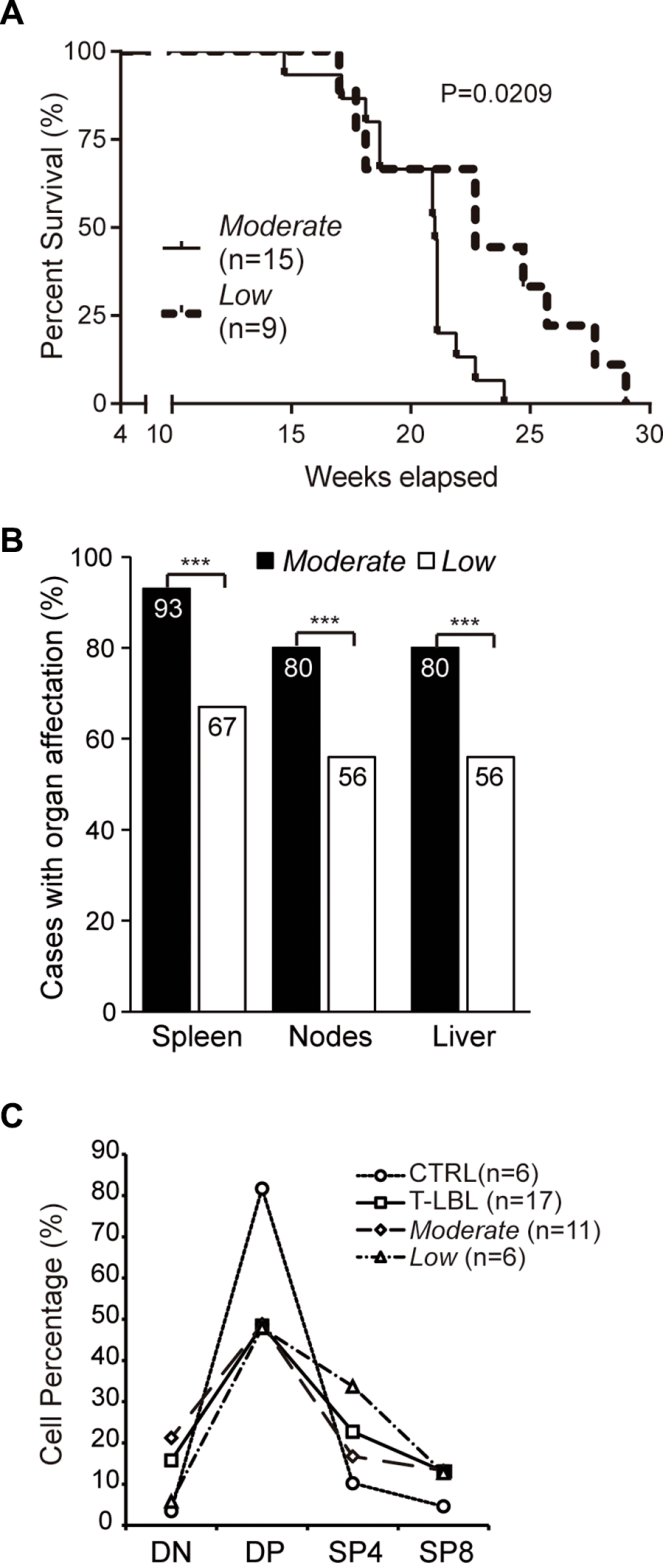
Aggressivenes of the “Moderate” and “Low” T-LBL sub-groups (**A**) Kaplan-Meier survival curve analysis comparing mice from the *Moderate* and *Low* T-LBL sub-groups. (**B**) Involvement of secondary organs in mice from the *Moderate* and *Low* T-LBL sub-groups, expressed in a bar chart as the percentage of cases with organ affectation. The statistical significance of the comparisons is indicated. (**C**) Thymocyte populations content, in percentage, in healthy thymuses (CTRL), the whole T-LBL series (T-LBL), and the *Moderate* and *Low* sub-groups. DN, CD4^−^CD8^−^ (*double-negative*). DP, CD4^+^CD8^+^ (*double-positive*). SP4, CD4^+^CD8^−^ (*single-positive CD4*). SP8, CD4^−^CD8^+^ (*single-positive CD8*). ***P ≤ 0.001.

A Kaplan-Meier survival curve analysis revealed that the median survival time was significantly longer for mice from the *Low* T-LBL sub-group, compared with that of the *Moderate* sub-group (22.7 *vs*. 21.0 weeks; Log-rank (Mantel-Cox) test, *P* = 0.0209) (Figure 4A). According to the Ann Arbor staging system, another measure of aggressiveness is the involvement of extralymphatic organs [30]. Very interestingly, the percentages of mice presenting hypertrophic spleen, nodes (either axillar, inguinal or mesenteric) and liver were significantly higher in the *Moderate* than in the *Low* sub-group (*P <* 0.001) (Figure 4B).

### Thymocyte developmental stages are differentially affected in “Moderate” and “Low” T-LBLs

The thymocyte populations content was determined by flow cytometry in the T-LBL samples, in comparison with healthy thymuses (Figure 4C). The T-LBL series exhibited a 1.7-fold-decrease in the CD4^+^CD8^+^ population (*double-positive*, DP) (mean ± SEM, 48.5% ± 7.02), in comparison with the control group (81.7% ± 0.63); this occurred in detriment of the other populations, with the highest increment in the CD4^−^CD8^−^ (*double-negative*, DN) subset, with a 4.5-fold-increase in tumors (3.5% ± 0.17 in controls *vs*. 15.8% ± 4.96 in T-LBLs).

Interestingly, the most prominent decrease of the DP population observed in the T-LBL series occurred to the same extent in both the *Moderate* (48.8% ± 9.09) and *Low* (47.9 ± 11.98) sub-groups. The concomitant increase in the DN population exhibited, however, a different behavior in the two sub-groups, with a level similar to that of the healthy thymuses in the *Low* sub-group (5.8% ± 2.40), but higher in the *Moderate* sub-group (21.2% ± 7.13) (6.1-fold-increase).

### FADD phosphorylation regulators in mouse T-LBL

**Figure 5:**
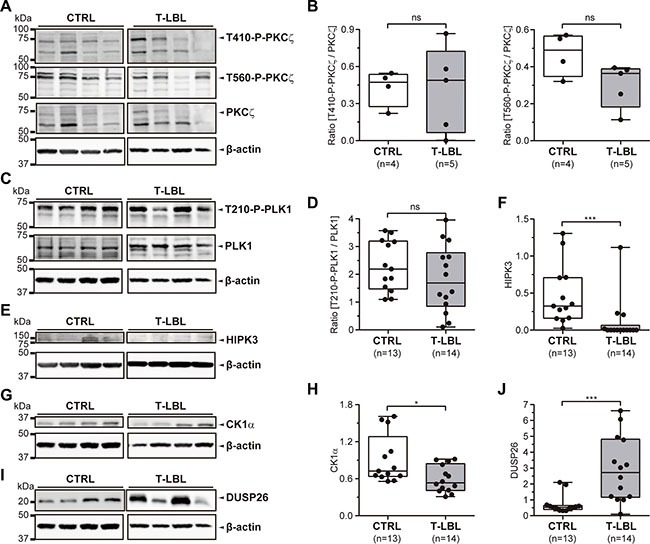
FADD phosphorylation regulators in T-LBL (**A**, **B**) PKCζ, T410-P-PKCζ, T560-P-PKCζ, PLK1, T210-P-PLK1, HIPK3, CK1α and DUSP26 levels were determined in healthy thymuses (CTRL) and T-cell lymphoblastic lymphoma samples (T-LBL) by Western blot. (A, **C**, **E**, **G**, **I**) Representative images are shown. WB images are cropped in favor of conciseness. (B, **D**, **F**, **H**, **J**) The box-and-whisker plot analyses of the ratios [T410-P-PKCζ/PKCζ], [T560-P-PKCζ/PKCζ], [T210-P-PLK1/PLK1], and the levels of HIPK3, CK1α and DUSP26 for all the samples are shown, indicating the statistical significance of the comparisons. *P < 0.05. ***P ≤ 0.001.

Several putative regulators of FADD phosphorylation have been proposed: the kinases HIPK3, PKCζ, CK1α, PLK1, and the phosphatase DUSP26. We analyzed all of them by Western blot in control and T-LBL samples from the same series used for the analysis of FADD and S191-P-FADD, by means of specific detection of PKCζ, T410-P-PKCζ, T560-P-PKCζ, PLK1, T210-P-PLK1, HIPK3, CK1α and DUSP26 (Figure 5).

The statistical analysis after densitometry and β-actin normalization revealed no significant alteration for PKCζ levels or its phosphorylated (active) forms in the controls *versus* T-LBLs comparison, as shown by the ratios [T410-P-PKCζ/PKCζ] and [T560-P-PKCζ/PKCζ] (*P* = 1.000 and *P* = 0.111, respectively) (Figure 5A, 5B). Likewise, the ratio [T210-P-PLK1/PLK1] did not change significantly between controls and T-LBLs (*P* = 0.325) (Figure 5C, 5D). Using molecules reported to inhibit PKCζ (Gö6976) [31] and PLK1 (GW842682X) [32] kinase activities, we did not observe any effect on FADD phosphorylation either in human (JURKAT) or murine (BW5147.3) T-LBL/ALL cell lines (Supplementary Figure S1).

**Figure 6:**
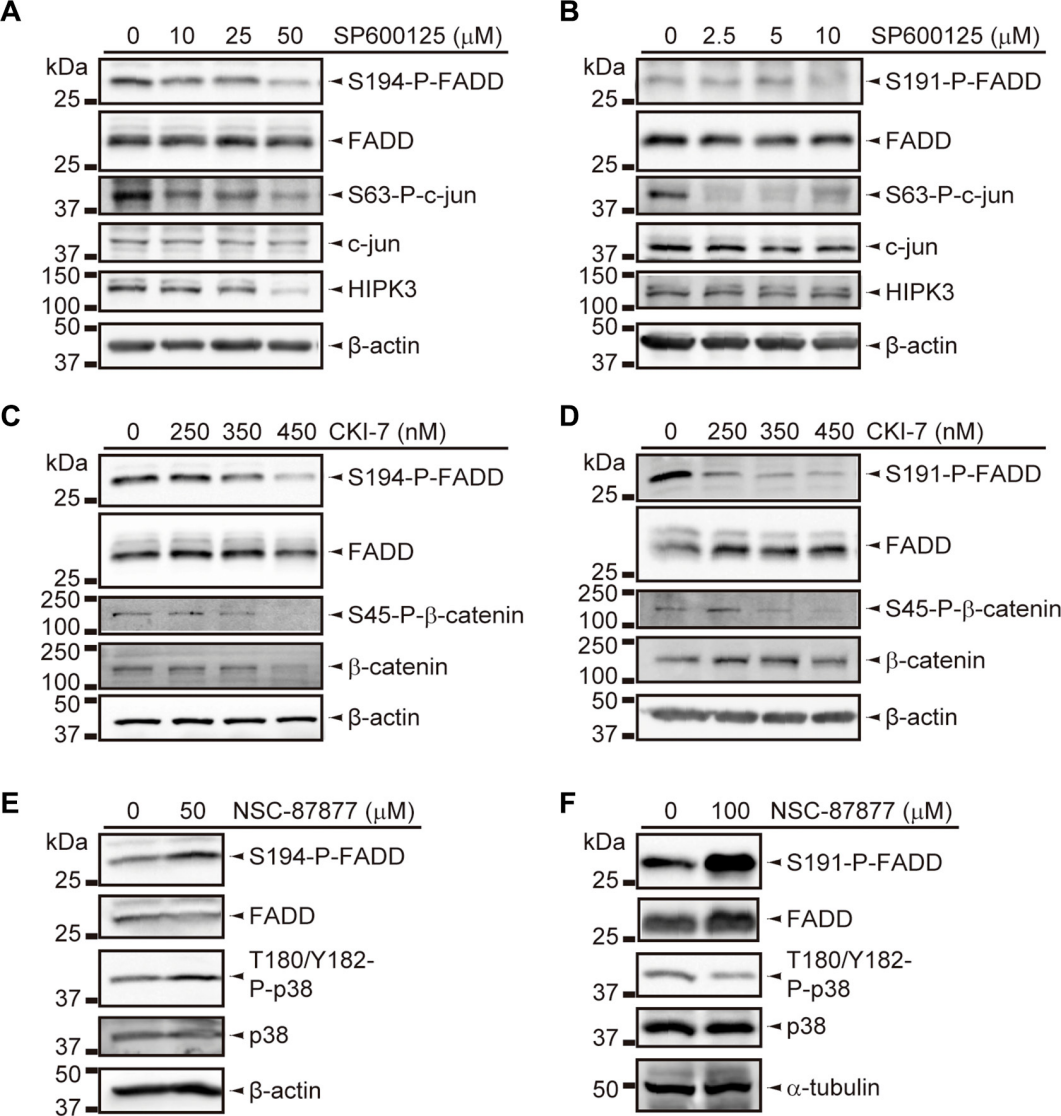
Inhibition assays for FADD phosphorylation regulators (**A**, **B**) Decrease of FADD phosphorylation in JURKAT (A) and BW5147.3 (B) cell lines, upon inhibition of HIPK3 by SP6000125-treatment for 5 h and 2 h, respectively, and the indicated doses. Inhibition was validated based on decrease in S63-P-c-jun and HIPK3 levels. (**C**, **D**) Decrease in FADD phosphorylation in JURKAT (C) and BW5147.3 (D) cell lines, upon inhibition of CK1α by CKI-7-treatment for 12 h and 8 h, respectively, and the indicated doses. Inhibition was validated based on decrease in S45-P-β-catenin level. (**E**, **F**) Increase of FADD phosphorylation in JURKAT (E) and BW5147.3 (F) cell lines, upon inhibition of DUSP26 by NSC-87877-treatment for 3 h, followed by 30 min-incubation of cells with 1 mM H_2_O_2_, and the indicated doses. Inhibition was validated based on increase of T180/Y182-P-p38 levels in JURKAT, but this effect could not be observed in BW5147.3. All WB images are cropped in favor of conciseness.

We found significant reductions of HIPK3 and CK1α expression levels in T-LBL samples (*P <* 0.001 and *P* = 0.012, respectively), together with a significant increase of DUSP26 (*P ≤* 0.001) (Figure 5E–5J). Then we sought to corroborate the role of these elements as *bona fide* regulators, especially as their activation levels could not be evaluated by specific detection of active forms. No specific inhibitors are available for HIPK3, but specific inhibition of JNK activity using SP600125 leads to a concomitant decrease in HIPK3 expression and FADD phosphorylation [21]. Using this inhibitor, we found a dose-dependent reduction of S194/S191-P-FADD in JURKAT and BW5147.3 cell lines (Figure 6A, 6B). Likewise, a dose-dependent reduction of S194/S191-P-FADD was observed in JURKAT and BW5147.3 cell lines after pharmacological inhibition of CK1α with a molecule reported to suppress its kinase activity, CKI-7 [26] (Figure 6C, 6D). Finally, we observed an increase of FADD phosphorylation after inhibition of DUSP26 phosphatase with the molecule NSC-87877, known to suppress its enzymatic activity [27] (Figure 6E, 6F).

## DISCUSSION

Changes in FADD expression and post-translational modification in cancer might be cell type-specific, thus resulting either in loss of apoptosis or gain of non-apoptotic functions [33, 34]. This would explain apparent discrepancies between studies. For example, FADD overexpression and increased phosphorylation correlate with poor clinical outcome and reduced patient survival in human lung adenocarcinoma [34]. More recently, FADD expression and phosphorylation were reported to be pro-tumorigenic in oncogenic KRAS-driven cancers [26]. However, mutations of the Ras family are relatively rare in T-cell acute lymphoblastic leukemia [35]. Collected evidence from COSMIC database corroborate that mutations in *KRAS* are not frequent in hematological malignancies, with a general incidence of 4.92%, which is even lower in T-ALL/LBL (1.52%) (http://cancer.sanger.ac.uk/cosmic).

On the other hand, FADD reduction has been reported in different tumor types [6–8]. A study on acute myeloid leukaemia [36] reported that reduced FADD in leukaemic cells at diagnosis indicated bad prognosis. It has been postulated that, in those many studies in which tumor progression has been shown to correlate with reduced FADD or phospho-FADD, the apoptotic role of FADD is stressed, and thus is FADD regarded as a tumor suppressor gene [9, 36].

We report here that FADD is significantly reduced in murine T-LBL samples. We discarded the upregulation of several microRNAs targeting *Fadd* as a mechanism underlying FADD decrease in T-LBL (data not shown). However, we find a very significant reduction of the putative transcription factor Ahr, which prompts us to suggest that significant downregulation of *Ahr* in T-LBLs may be involved in FADD reduction in these samples.

However, the ability of FADD as a “proliferation-apoptosis coupler” [20] introduces an additional level of complexity. Not only FADD reduction may contribute to cancer, but also its phosphorylation status would determine its proliferative role. Additionally, FADD has a critical role in thymopoiesis, which potentially makes T-LBL a type of cancer particularly susceptible to FADD alterations.

FADD phosphorylation has been analyzed in numerous solid tumors, for example in prostate cancer cells, where a reduction of FADD phosphorylation has been reported [37, 38]. These and other authors suggest that assessment of FADD phosphorylation may be useful as a prognostic biomarker, and that induction of FADD phosphorylation could be a target in cancer therapy [39]. However, very limited results regarding FADD phosphorylation are available in hematological human cancers [29]. We report here that murine T-LBL samples exhibit a pronounced reduction of S191-phospho-FADD, thus resulting the ratio [S191-P-FADD/FADD] considerably diminished. However, a considerable inter-tumor heterogeneity was observed. A similar scenario has been previously reported in T-cell tumors and B-cell non-Hodgkin lymphoma, where both phospho-FADD positivity and signal intensity exhibited a high variation [28, 29].

We establish two T-LBL sub-groups (*Moderate* and *Low*), according to S191-P-FADD positivity determined by IHC. Parameters of tumor aggressiveness like survival time and secondary organ involvement support our stratification in *Moderate* and *Low* T-LBL sub-groups.

Cytoplasmic FADD positivity exhibits a significant reduction in T-LBL cells from both groups, consistent with a reduced participation of FADD in apoptosis. On the other hand, S191-P-FADD positivity exhibits a reduction particularly striking in the nucleus of thymocytes belonging to the *Low* T-LBL group. Since the proliferation rate of this group is significantly lower than that of the *Moderate* T-LBL group, this is consistent with a role for FADD in functions other than apoptosis, like proliferation. This is in agreement with previous observations associating Ki67 and phospho-FADD in human lung adenocarcinoma [34] and B-cell non-Hodgkin lymphoma [28].

Since early T cell development in FADD-deficient mice is inhibited at the DN to DP transition [13], we suggest that DP reduction in murine T-LBLs might be explained, at least in part, based on FADD decrease. On the other hand, the differential accumulation of DN cells in tumors from the *Moderate* and *Low* sub-groups suggests that FADD might intervene in the differentiation and proliferation of tumor thymocytes, but these processes would be uncoupled.

Regarding FADD phosphorylation, various regulators may operate. Classical cell cycle-regulating kinases such as cdc and CDKs, as well as PKCs, have been discarded [40]. More specifically, it has been reported that PKCζ does not phosphorylate FADD in DU145 prostate cancer cells [21]. This is in agreement with our results in T-LBL, where no alterations in PKCζ activation were observed, nor any effect on FADD phosphorylation upon pharmacological inhibition in T-LBL/ALL cell lines. We obtained similar results for PLK1. We found, however, that HIPK3 and CK1α kinases exhibit a significant reduction in T-LBLs, together with a significant increase of the phosphatase DUSP26. We suggest that these enzymes might co-operate in regulating FADD phosphorylation in thymocytes, and that their concomitant deregulation might underlie the alterations we observe in T-LBLs.

**Figure 7:**
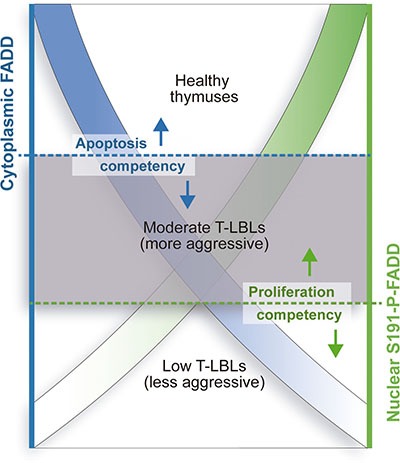
Model for differential aggressiveness of the T-LBL sub-groups Two different thresholds for cytoplasmic FADD level (left axis, in blue) and nuclear S191-P-FADD level (right axis, in green) in T-LBL samples define the limits for apoptosis and proliferation competency, respectively. T-LBL samples with cytoplasmic FADD levels below the *blue* threshold are not able to undergo FADD-dependent apoptosis, which may characterize them as tumors. However, the *green* threshold for nuclear S191-P-FADD level determines the capacity of the tumor cells to undergo FADD-dependent proliferation, provided that FADD gets phosphorylated and properly distributed to the nucleus. Thus, those samples between the two thresholds, the *Moderate* T-LBL sub-group, would present with a more aggressive phenotype than those below both of them, the *Low* T-LBL sub-group. Accordingly, a healthy thymus would be balanced in terms of FADD-mediated apoptosis and proliferation, with FADD levels enabling them for both functions.

We propose a model of double-threshold (Figure 7), where the level of cytoplasmic FADD would define its canonical function as an apoptotic adapter and the level of nuclear S191-P-FADD would define its participation in proliferative, cell cycle control or survival non-canonical processes. According to this model, tumor cells within the window between the two thresholds (*Moderate* T-LBLs) would lack proper FADD-involved apoptosis, but would be competent for FADD-mediated proliferation, cell cycle and/or survival, thus becoming more aggressive. Control cells would be balanced in terms of FADD-mediated apoptosis and proliferation, with FADD levels enabling them for both functions, whereas tumor cells from the *Low* sub-group would be incompetent both for FADD-mediated apoptosis and proliferation, thus resulting in less aggressive tumors.

In summary, our data indicate that T-LBL cells exhibit a reduction of FADD availability in the cytoplasm, which may contribute to impaired apoptosis. But also, our results indicate that different statuses of FADD phosphorylation may be a key element providing the basis for tumor stratification. The resultant balance between FADD-dependent apoptotic and proliferative capacities may define the outcome of the tumor. With this study, we gain knowledge on the status, regulation and functionality of FADD in T-LBL, which will help establish a better prognosis and contribute to develop new therapeutic strategies.

## MATERIALS AND METHODS

### Murine samples

The experiments with primary tumors were performed using a mouse model of T-LBL. We adhered to the ethical considerations dictated by the European Directive 2010/63/EU and Real Decreto 53/2013 on the protection of animals used in scientific procedures. 4-week-aged C57BL/6J female mice obtained from The Jackson Laboratory (The Jackson Laboratory, Bar Harbor, ME, USA) were either left untreated (healthy thymuses, control group) or subjected to T-cell lymphoblastic lymphoma (T-LBL group) induction as described in [41]. A portion of the thymuses (control or T-LBL) was mechanically dispersed and strained through a nylon mesh (BD Biosciences, San Jose, CA, USA) to isolate the thymocytes. Another portion was fixed for Immunohistochemistry.

### Cell culture

JURKAT cell lines A3 (ATCC^®^ CRL-2570^™^) and I 2.1 (ATCC^®^ CRL-2572^™^), and BW5147.3 (Thy-1-e) cell line (ATCC^®^ TIB-234^™^) were purchased from the American Type Culture Collection (ATCC, Manassas, VA, USA). ATCC routinely performs cell lines authentication, using STR analysis (DNA profiling) as a procedure. Cell experimentation was always performed within a period not exceeding 6 months after resuscitation. Cells were cultured in RPMI 1640 (Gibco, Grand Island, NY, USA) supplemented with 10% or 15% FBS (PAA Laboratories, GE Healthcare Life Sciences, Velizy-Villacoublay, France), 2 mM L-Glutamine (Merck Millipore, Billerica, MA, USA) and 1 mM sodium pyruvate (Merck Millipore). Cultures were maintained at 37°C in 5% CO_2_ humidified atmosphere.

### Gene expression analysis

Total RNA from murine samples was obtained using TriPure Reagent (Roche Applied Science, Indianapolis, IN, USA), following manufacturer's instructions. *Fadd* and *Ahr* gene expressions were determined at the transcriptional level by real-time quantitative RT-PCR from total RNA in two steps, using first the High-Capacity RNA-to-cDNA™ Kit (Applied Biosystems, Foster City, CA, USA), then the FastStart Universal SYBR Green Master (Rox) (Roche, Mannheim, Germany). Expression values of *G6pd* and *Hprt* in the same samples were used for normalization, using the 2^−ΔΔC^_T_ method [42]. Primers are indicated in Supplementary Table S1.

### Fadd cDNA and fadd promoter amplification and sequencing

One microgram of RNA from T-LBL samples was reverse-transcribed using SuperScript^®^ VILO^™^ cDNA Synthesis Kit (Invitrogen, Carlsbad, CA, USA), and *Fadd* cDNA was amplified by PCR using Expand High Fidelity PCR System (Roche Applied Science) at T(annealing) = 55°C, with the primers indicated in Supplementary Table S1.

Genomic DNA from T-LBL samples was obtained using TriPure Reagent (Roche Applied Science), following manufacturer's instructions. *Fadd* promoter was amplified by PCR using GC-Rich PCR System (Roche Applied Science) at T(annealing) = 59.8°C and 0 M Resolution Solution, with the primers indicated in Supplementary Table S1.

The PCR products were purified using Wizard SV Gel and PCR Clean-up System (Promega Corporation, Madison, WI, USA). Sequencing reactions were performed using either an ABI Prism 310 Automated Sequencer or an 3730XL ABI Sequencer, from Applied Biosystems (Applied Biosystems, Thermo Fisher, Waltham, MA, USA), and the primers indicated in Supplementary Table S1. For comparisons, the program L-Align from ExPASy Molecular Biology Server was used.

### Antibodies and reagents

Primary and secondary antibodies used for immunodetection are summarized in Supplementary Table S2. Gö6976, SP600125 and NSC-87877 were purchased from Calbiochem (Merck Millipore), CKI-7 from Sigma-Aldrich (Sigma-Aldrich, St. Louis, MO, USA) and GW843682X from Tocris Bioscience (Tocris Bioscience, Bristol, United Kingdom).

### Western blot

Total proteins from murine samples were obtained using TriPure Reagent (Roche Applied Science), following manufacturer's instructions. Total proteins from cell lines were obtained using radioimmunoprecipitation assay (RIPA) cell lysis buffer. Protein extracts were supplemented with 2 mM phenylmethylsulphonyl fluoride (PMSF), 2.5 μl/ml Protease Inhibitor Cocktail and 10 μl/ml Phosphatase Inhibitor Cocktail 2 (Roche Diagnostics GmbH, Mannheim, Germany). Ten micrograms-aliquots were electrophoresed in 12% SDS-PAGE with β-mercaptoethanol, then electrotransferred to Immobilon-P transfer membranes (Merck Millipore).

The peroxidase activity was developed using WesternBright ECL Detection System (Advansta, Menlo Park, CA, USA). ImageQuant LAS 4000 digital imaging system (GE Healthcare Bio-Sciences, Piscataway, NJ, USA) was used for acquisition of images, and Scion Image Software (Scion Corporation, NIH, Frederick, MD, USA) for band densitometry.

### Immunohistochemistry

Formalin-fixed and embedded paraffin tissues were deparaffinised and rehydrated using standard protocols and subjected to heat-induced antigen retrieval in Tris-EDTA buffer (pH = 9.0).

Frozen tissues were obtained after 10% formalin-fixation for 2 h, cryoprotection with 30% sucrose in phosphate buffer saline (PBS) for 24 h, and embedding in Tissue-Tek^®^ O.C.T ™ Compound (Sakura Finetek Europe B.V., Alphen aan den Rijn, The Netherlands). 10 μm-sections were used.

Endogenous peroxidase activity was reduced by 3% H_2_O_2_-pre-treatment for 30 min. Dako REAL^™^ antibody diluent (Dako, Glostrup, Denmark) was the blocking buffer. We used liquid DAB+ substrate chromogen system (Dako) for visualization. The sections were counterstained with Mayer's hematoxylin (Sigma-Aldrich). An Axiovert 200 inverted microscope (Carl Zeiss, Oberkochen, Germany) and a SPOT RT Digital Scanning Camera (Diagnostic Instruments, Sterling Heights, MI, USA) at 63 × magnification were used for image analysis. Photographic material was acquired with an Olympus BX61 microscope (Olympus America, Melville, NY, USA) at 20 × and 100 × magnification. Two authors evaluated immunostaining independently. Irrespective of intensity, the percentages of positive cells *versus* total cell number were calculated using ten random representative fields per section. Fiji-Image J free software (http://fiji.sc; National Institutes of Health, Bethesda, MD, USA) was used for analysis, applying the *Image/Colour deconvolution*, *Image/Adjust/Threshold* and *Analyze/Analyze Particles* tools as published previously [43].

### *In vitro* FADD reconstitution

I 2.1 FADD-deficient JURKAT cells were electroporated with EX-V0108-Lv225 or EX-NEG-Lv225 constructs (GeneCopoeia, Rockville, MD, USA) using a Gene Pulser MXcell Electroporation System (Bio-Rad Laboratories, Hercules, CA, USA). 10^7^ cells in 500 μl of complete medium were subjected to 280V, 950 μF, with 5 μg or 25 μg of vector. Electroporated cells were harvested in 10 ml of complete medium and analyzed for protein expression 48 h post transfection, both by flow cytometry - testing GFP positivity with a FACS Canto II (Becton-Dickinson, Franklin Lakes, NJ, USA) for percentage and mean fluorescence intensity - and Western blot. Then, 2 × 10^5^ cells at 10^6^ cells/ml were treated for 24 h with agonist anti-FAS mouse monoclonal antibody (clone CH11; Merck Millipore) or mouse IgM λ isotype control (clone 11E10; Beckman Coulter, Nyon, Switzerland), and aliquoted for (1) protein extraction and WB and for (2) Annexin V/7-AAD apoptosis assay by flow cytometry using PE Annexin V Apoptosis Detection Kit I (BD-Pharmingen™) and a FACSCalibur flow cytometer (Becton-Dickinson). In parallel, untreated transfected cells were followed for cell growth, based on Trypan blue exclusion viability test.

### Flow cytometry analysis

Freshly isolated thymocytes from healthy thymuses and T-LBL samples were examined by flow cytometry. A CD3/TdT/CD4/CD8 4-colour analysis was performed on a FACSCalibur flow cytometer (BD Biosciences). 25 000 cells were analyzed and background levels were determined with isotype-matched control antibodies (Supplementary Table S2).

Data analyses were performed using FlowJo (Flowjo, LLC, OR, USA).

### Statistical analyses

Significances were determined by a Mann-Whitney *U* test and a Bonferroni correction using the Statistical Package for the Social Sciences software (SPSS v.23.0, IBM Corporation, Somers, NY, USA). Fisher's exact test was used to compare the distribution of two categories. Statistical analysis of Kaplan-Meier survival curves was performed by the Mantel-Cox method, using GraphPad Prism 6 (GraphPad Software, La Jolla, CA, USA). Kernel density plot was performed with R free software (R Development Core Team, version 3.2.3).

## SUPPLEMENTARY MATERIALS FIGURES AND TABLES




