# Supplementary material for: Deregulated FADD expression and phosphorylation in T-cell lymphoblastic lymphoma
Source: Oncotarget. 2016 Aug 18;7(38):61485–99. doi: 10.18632/oncotarget.11370 (PMC5308666; doi:10.18632/oncotarget.11370)
Supplement: Supplementary file 1 [file oncotarget-07-61485-s001.pdf]

Deregulated FADD expression and phosphorylation in T-cell lymphoblastic lymphoma

Supplementary Materials

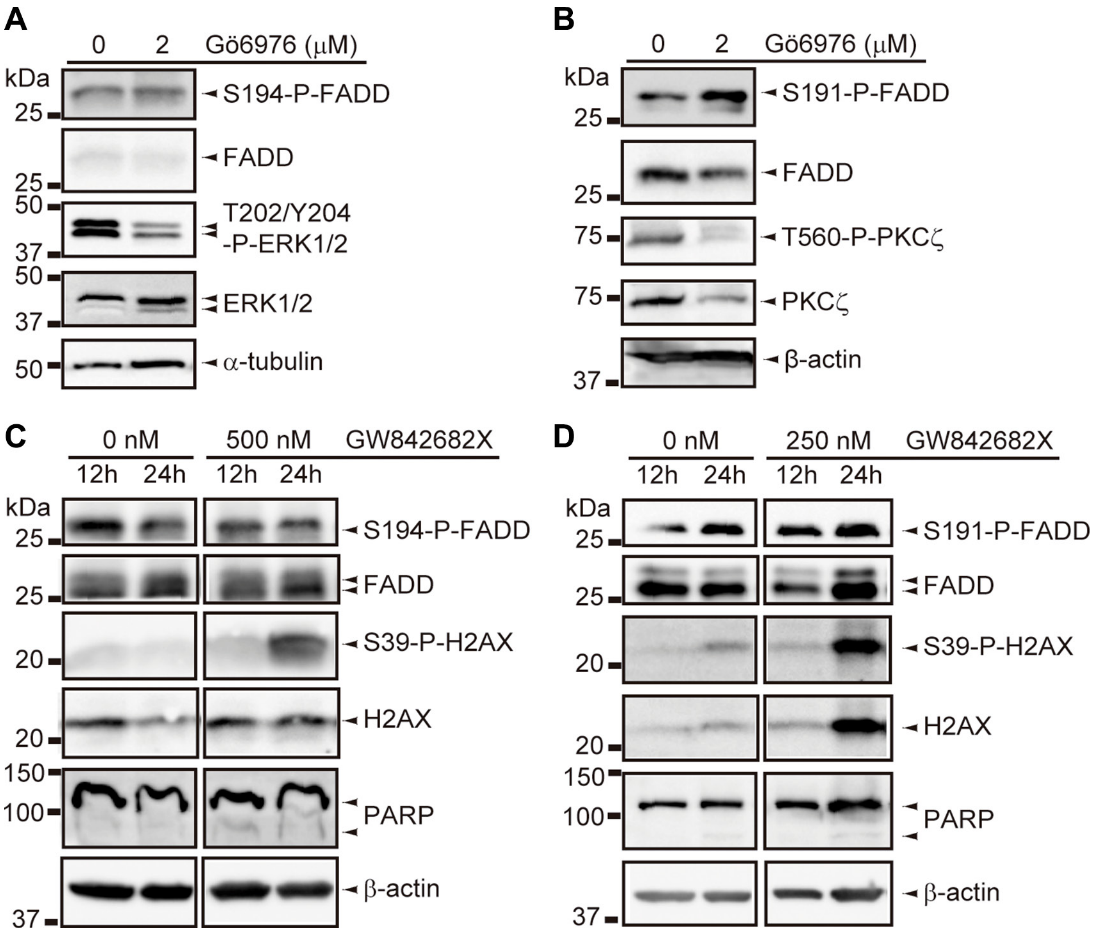

Supplementary Figure S1: Inhibition assays for PKCζ and PLK1. (**A**, **B**) Assessment of FADD phosphorylation in JURKAT (A) and BW5147.3 (B) cell lines, upon inhibition of PKCζ by Gö6976-treatment for 12 h and 6 h, respectively, and the indicated doses. Inhibition was validated based on decrease of T202/Y204-P-ERK1/2 (A) or T560-P-PKCζ levels (B). (**C**, **D**) Assessment of FADD phosphorylation in JURKAT (C) and BW5147.3 (D) cell lines, upon inhibition of PLK1 by GW842682X-treatment for 12 h and 24 h and the indicated doses. Inhibition was validated based on increase in S139-P-H2A.X level, together with PARP proteolysis. All WB images are cropped in favor of conciseness.

Supplementary Table S1: List of primers

Application	Gene/Target	Name	Sequence (5′–3′)
qPCR	*Fadd*	Forward	CGCCGACACGATCTACTGCA
		Reverse	CAGACACCTTCAGCTCGCGG
	Ahr	Forward	GCACAAGGAGTGGACGAAG
		Reverse	AGGAAGCTGGTCTGGGGTAT
	G6pd	Forward	ACGACATCCGAAAGCAGAGT
		Reverse	CATAGGAATTACGGGCAAAGA
	Hprt	Forward	TCCTCCTCAGACCGCTTTT
		Reverse	CCTGGTTCATCATCGCTAAT
PCR	*Fadd cDNA*	Forward	GGTACCGATTTCCTATGTGGGATCGC
		Reverse	CTCGAGTCCGGGTGTTTCTGAGGAAG
	Fadd Promoter	Forward	CAAAGCCCCTGGAACTGGAA
		Reverse	TTTACTCCACAGGCCCTCCAG
Sequencing	*Fadd cDNA*	Forward	TATGTGGGATCGCTGAGGGG
		Reverse	AGACACAGTTGAATCCCTTA
	Fadd Promoter	Forward-1	CCAGCCCATAAGTAACTCCATCCA
		Forward-2	CGCCCACTTTTTTCAACCGC
		Reverse-1	GCCCTCCAGCTGCTTTGATG
		Reverse-2	CAGTAGATCGTGTCGGCGCA

The table indicates the sequence of each primer used for quantitative PCR, standard PCR or sequencing of different genes or target sequences.

Supplementary Table S2: List of antibodies used for immunodetection. The table indicates the techniques and proteins detected with each antibody, and details on their origin and use. O/N, overnight. RT, room temperature. See Supplementary_Table_S2
