# Supplementary material for: Deregulated FADD expression and phosphorylation in T-cell lymphoblastic lymphoma
Source: Oncotarget. 2016 Aug 18;7(38):61485–99. doi: 10.18632/oncotarget.11370 (PMC5308666; doi:10.18632/oncotarget.11370)
Supplement: Supplementary file 2 [file oncotarget-07-61485-s002.doc]

**Supplementary Table S2. *List of antibodies used for immunodetection*.**

| ***Supplementary Table S2. List of antibodies used for immunodetection*** | | | | | |
| --- | --- | --- | --- | --- | --- |
| **Western Blot** | **Clone/Conjugation** | **Dilution** | **Incubation** | **Origin** | **Company** |
| **β-actin** | AC-15 | 1/20000 (Milk) | 4ºC O/N | Mouse | Cell Signaling Technology (Danvers, MA, USA) |
| **Caspase-3** | Polyclonal | 1/1000 (Milk) | 4ºC O/N | Rabbit | Cell Signaling Technology |
| **(D175)-cleaved Caspase-3** | Polyclonal | 1/1000 (Milk) | 4ºC O/N | Rabbit | Cell Signaling Technology |
| **Caspase-8** | 1C12 | 1/500 (BSA) | 4ºC O/N | Mouse | Cell Signaling Technology |
| **(D391)-cleaved Caspase-8** | 18C8 | 1/500 (BSA) | 4ºC O/N | Rabbit | Cell Signaling Technology |
| **β-catenin** | 14 | 1/1000 (Milk) | 4ºC O/N | Mouse | BD Biosciences (San Jose, CA, USA) |
| **S45-P-β-catenin** | Polyclonal | 1/1000 (BSA) | 4ºC O/N | Rabbit | Cell Signaling Technology |
| **CK1α** | EPR1961(2) | 1/2000 (Milk) | 4ºC O/N | Rabbit | Abcam (Cambridge, United Kingdom) |
| **DUSP26** | Polyclonal | 1/500 (Milk) | 4ºC O/N | Rabbit | Origene (Rockville, MD, USA) |
| **ERK1/2** | Polyclonal | 1/1000 (BSA) | 4ºC O/N | Rabbit | Cell Signaling Technology |
| **T202/Y204-P-ERK1/2** | Polyclonal | 1/1000 (BSA) | 4ºC O/N | Rabbit | Cell Signaling Technology |
| **FADD** | IF7 | 1/500 (Milk) | 4ºC O/N | Mouse | Merck Millipore |
| **S191-P-FADD** | Polyclonal | 1/1000 (BSA) | 4ºC O/N | Rabbit | Cell Signaling Technology |
| **S194-P-FADD** | Polyclonal | 1/500 (BSA) | 4ºC O/N | Rabbit | Cell Signaling Technology |
| **GAPDH** | ID4 | 1/1000 (Milk) | 4ºC O/N | Mouse | Enzo Life Sciences (Farmingdale, NY, USA) |
| **H2A.X** | Polyclonal | 1/2000 (BSA) | 4ºC O/N | Rabbit | Cell Signaling Technology |
| **S139-P-H2A.X** | Polyclonal | 1/1000 (BSA) | 4ºC O/N | Rabbit | Cell Signaling Technology |
| **HIPK3** | Polyclonal | 1/250 (Milk) | 4ºC O/N | Rabbit | Abcam |
| **c-Jun** | Polyclonal | 1/1000 (BSA) | 4ºC O/N | Rabbit | Cell Signaling Technology |
| **S63-P-c-Jun** | 54B3 | 1/1000 (BSA) | 4ºC O/N | Rabbit | Cell Signaling Technology |
| **p38** | Polyclonal | 1/1000 (BSA) | 4ºC O/N | Rabbit | Cell Signaling Technology |
| **T180/Y182-P-p38** | Polyclonal | 1/1000 (BSA) | 4ºC O/N | Rabbit | Cell Signaling Technology |
| **PARP** | A6.4.12 | 1/1000 (Milk) | 4ºC O/N | Mouse | AbD Serotec (Oxford, United Kingdom) |
| **PKCζ** | C24E6 | 1/1000 (Milk) | 4ºC O/N | Rabbit | Cell Signaling Technology |
| **T410/103-P-PKCζ/λ** | Polyclonal | 1/1000 (BSA) | 4ºC O/N | Rabbit | Cell Signaling Technology |
| **T560-P-PKCζ** | Polyclonal | 1/2000 (BSA) | 4ºC O/N | Rabbit | Abcam |
| **PLK1** | Polyclonal | 1/500 (Milk) | 4ºC O/N | Rabbit | Santa Cruz Biotechnology (Dallas, Texas, USA) |
| **T210-P-PLK1** | EPNCIR167 | 1/1000 (BSA) | 4ºC O/N | Rabbit | Abcam |
| **α-Tubulin** | DM1A | 1/10000 (Milk) | 4ºC O/N | Mouse | Sigma-Aldrich |
| **Anti-mouse IgG** | HRP-conjugated | 1/1000 (BSA) | 4ºC O/N | Horse | Cell Signaling Technology |
| **Anti-rabbit IgG** | HRP-conjugated | 1/1000 (BSA) | 4ºC O/N | Goat | Cell Signaling Technology |
| **Immunohistochemistry** | **Clone/Conjugation** | **Dilution** | **Incubation** | **Origin** | **Company** |
| **(D175)-cleaved Caspase-3** | Polyclonal | 1/100 | 4ºC O/N | Rabbit | Cell Signaling Technology |
| **FADD** | Polyclonal | 1/100 | 4ºC O/N | Rabbit | Origene |
| **S191-P-FADD** | Polyclonal | 1/100 | 4ºC O/N | Rabbit | Santa Cruz Biotechnology |
| **Ki67** | SP6 | Ready-to-Use | RT 1h | Rabbit | Master Diagnostica (Granada, Spain) |
| **TdT** | Polyclonal | Ready-to-Use | RT 1h | Rabbit | Dako |
| **Anti-rabbit IgG** | HRP-conjugated | 1/50 | RT 1h | Goat | Dako |
| **Flow Cytometry** | **Clone/Conjugation** | **Dilution** | **Incubation** | **Origin** | **Company** |
| **CD3e** | 145-2C11 / FITC-conjugated | 1/100 | 4ºC 30 min | Hamster | BD Biosciences |
| **CD4** | RM4-5 / APC-conjugated | 1/100 | 4ºC 30 min | Rat | BD Biosciences |
| **CD8a** | 53-6.7 / PerCP-conjugated | 1/50 | 4ºC 30 min | Rat | BD Biosciences |
| **TdT** | 19-3 / PE-conjugated | 1/100 | 4ºC 30 min | Mouse | eBiosciences (San Diego, CA, USA) |
| **IgG1, κ Isotype Control** | A19-3 / FITC-conjugated | 1/50 | 4ºC 30 min | Hamster | BD Biosciences |
| **IgG2a, κ Isotype Control** | R35-95 / PE-conjugated | 1/50 | 4ºC 30 min | Rat | BD Biosciences |
| **IgG2a, κ Isotype Control** | R35-95 / APC-conjugated | 1/50 | 4ºC 30 min | Rat | BD Biosciences |
| **IgG2a, κ Isotype Control** | R35-95 / PerCP-conjugated | 1/50 | 4ºC 30 min | Rat | BD Biosciences |
